# Identification of the Adult Hematopoietic Liver as the Primary Reservoir for the Recruitment of Pro-regenerative Macrophages Required for Salamander Limb Regeneration

**DOI:** 10.3389/fcell.2021.750587

**Published:** 2021-09-22

**Authors:** Ryan J. Debuque, Andrew J. Hart, Gabriela H. Johnson, Nadia A. Rosenthal, James W. Godwin

**Affiliations:** ^1^Australian Regenerative Medicine Institute, Monash University, Melbourne, VIC, Australia; ^2^The MDI Biological Laboratory (MDIBL), Kathryn W. Davis Center for Regenerative Biology and Aging, Salisbury Cove, ME, United States; ^3^The Jackson Laboratory, Bar Harbor, ME, United States

**Keywords:** regeneration, macrophage, salamander, hematopoiesis, wound healing, leukocyte trafficking

## Abstract

The lack of scar-free healing and regeneration in many adult human tissues imposes severe limitations on the recovery of function after injury. In stark contrast, salamanders can functionally repair a range of clinically relevant tissues throughout adult life. The impressive ability to regenerate whole limbs after amputation, or regenerate following cardiac injury, is critically dependent on the recruitment of (myeloid) macrophage white blood cells to the site of injury. Amputation in the absence of macrophages results in regeneration failure and scar tissue induction. Identifying the exact hematopoietic source or reservoir of myeloid cells supporting regeneration is a necessary step in characterizing differences in macrophage phenotypes regulating scarring or regeneration across species. Mammalian wounds are dominated by splenic-derived monocytes that originate in the bone marrow and differentiate into macrophages within the wound. Unlike mammals, adult axolotls do not have functional bone marrow but instead utilize liver and spleen tissues as major sites for adult hematopoiesis. To interrogate leukocyte identity, tissue origins, and modes of recruitment, we established several transgenic axolotl hematopoietic tissue transplant models and flow cytometry protocols to study cell migration and identify the source of pro-regenerative macrophages. We identified that although bidirectional trafficking of leukocytes can occur between spleen and liver tissues, the liver is the major source of leukocytes recruited to regenerating limbs. Recruitment of leukocytes and limb regeneration occurs in the absence of the spleen, thus confirming the dependence of liver-derived myeloid cells in regeneration and that splenic maturation is dispensable for the education of pro-regenerative macrophages. This work provides an important foundation for understanding the hematopoietic origins and education of myeloid cells recruited to, and essential for, adult tissue regeneration.

## Introduction

Salamanders are the only vertebrate to regenerate limbs as adults, a trait that is lost in mammals and related amphibians (Godwin and Rosenthal, 2014). Adult scar-free repair and regeneration is restricted to very few vertebrate species (Tanaka and Reddien, 2011; Godwin, 2014; Godwin and Rosenthal, 2014). Understanding the molecular basis for the remarkable ability of the salamander to regenerate after extensive organ damage or tissue loss provides opportunities for future therapies aimed at enhancing human repair. The limb has a long history of gaining scientific attention as it is an accessible, morphologically complex structure that progresses through definable stages of progenitor cell activation, regrowth, and patterning (Tanaka, 2016). We previously identified that limb regeneration is critically dependent on early myeloid blood cell recruitment during a period when salamander myeloid cells share similar recruitment kinetics observed in mammalian tissue injuries (Mirza et al., 2009; Godwin et al., 2013; Aurora et al., 2014). Myeloid cells in mammals show considerable heterogeneity in phenotype and origin. Various macrophage subpopulations can play opposing roles in both repair and fibrosis within various contexts [reviewed in Godwin et al. (2017b)]. Identifying the origin, tissue reservoir, and mode of migration of myeloid cells participating in regeneration in the adult salamander will provide insights on how successful regeneration is mediated in adult tissues.

In mammals, the bone marrow is the major site of adult hematopoiesis, with the spleen also serving as an extramedullary site for monocyte production in some circumstances (Swirski et al., 2009). In salamanders, the bone marrow appears non-hematopoietic (Brunst, 1958; Durand et al., 2000; Golub et al., 2004). Recent reports have supported historical observations (Hightower and Haar, 1975) that the axolotl liver, spleen, and thymus serve as leukocyte niches similar to mammals, with the liver and spleen also acting as sites for hematopoiesis (Lopez et al., 2014). Understanding the leukocyte proportions in these tissues as well as in circulation during homeostasis is of great interest. In the past, tritiated thymidine has been used to label the newt liver and spleen prior to amputation (Hay and Fischman, 1961). Blood cells originating from both tissues are reported to migrate to the amputated limb but the extent of their contribution as well the identity of these infiltrating cells is unknown. To address this, we have opted to use modern flow cytometry approaches utilizing antibodies and lectins to enumerate distinct leukocyte subsets. In addition, we have developed a novel adult transgenic hematopoietic tissue transplantation model to study cellular migration from these niches during homeostasis and limb regeneration. Contrary to previous reports, which suggest that the adult spleen is the primary tissue that deploys leukocytes into the periphery (Lopez et al., 2014), we demonstrate that the liver is the main source of myeloid cells trafficking to distant injury sites such as the early regenerating tail or limb and can do so without passing through or requiring education within the spleen. These studies form a foundation for understanding the immune cell requirements for adult tissue regeneration.

## Results

### The Axolotl Peripheral Immune System Has a Profile Rich in B Cells and Granulocytes With Monocyte/Macrophage Numbers Typical of Other Species

**FIGURE 1:**
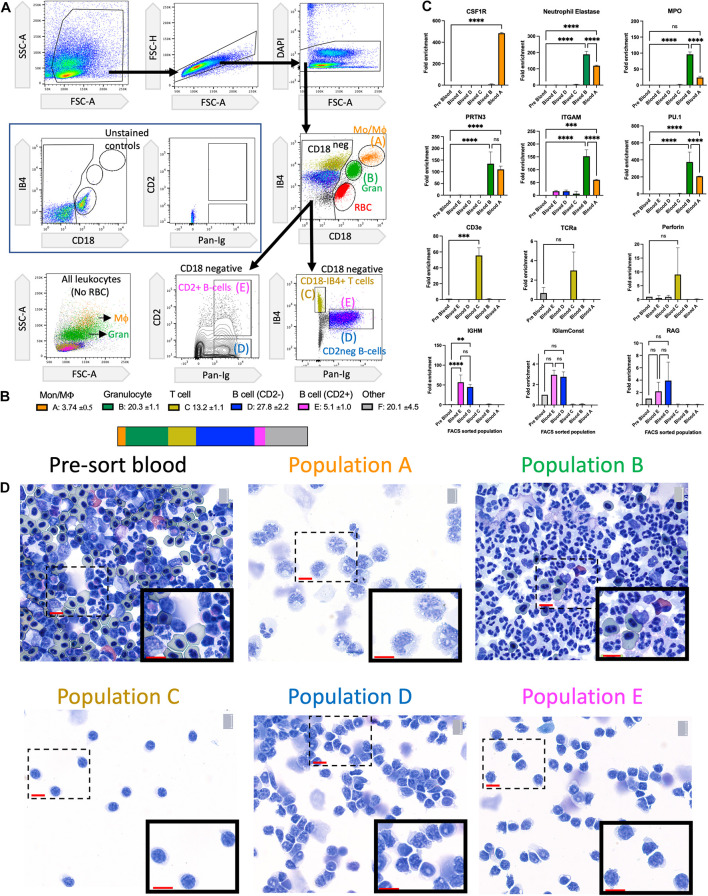
Identification of major leukocyte subsets in the axolotl peripheral circulation. **(A)** Flow cytometry gating strategy to identify viable circulating single-cell leukocytes in adult axolotls. Use of anti-CD18 and IB4 lectin to identify Monocyte/Macrophage (orange), Granulocytes (green), and RBCs (red). Use of the anti-Pan-Ig, anti-CD2, and IB4 lectin to identify T cells as well as two subsets of B cells. Populations are labeled A–E. **(B)** Enumeration and visualization of approximate cell subset frequency within total leukocyte population pool. The mean % of non-RBC shown for each population and SD is based on four biological replicates. **(C)** RT-PCR validation of population identity within FACS sorted populations A–E using representative cell-specific genes. Fold enrichment is calculated relative to pre-sorted blood. Significance calculated using two-way ANOVA with Turkey’s multiple comparisons test indicated as ^*ns*^*p* > 0.05, ^∗∗^*p* ≤ 0.01, ^∗∗∗^*p* ≤ 0.001, and ^****^p ≤ 0.0001 **(D)** Cytospins of FACS sorted populations A–E and pre-sort blood stained with Wright–Giemsa. Each population shows a high level of purity. Population A shows monocyte/macrophage morphology. Population B shows typical granulocyte morphology with multilobed nucleus. Populations C–E display typical lymphocyte morphology. 40 × magnification with scale bar = 20 microns. Mo/Mϕ, monocyte/macrophage; Gran, granulocyte; RBC, red blood cells.

We previously described the requirement for salamander macrophages in the success of limb regeneration (Godwin et al., 2013) and a basic flow cytometric toolkit for profiling phagocytic macrophages in the blood and regenerating limb (Debuque and Godwin, 2015; Debuque et al., 2021). To gain further insights into the recruitment of different axolotl immune cells to the regenerating limb and tail, we extended our flow cytometric panel to label additional non-myeloid cells. Deploying this more advanced panel allowed the identification of monocytes, macrophages, T cells, and several B cell subsets. Red blood cells (RBCs) in salamanders can present a considerable impediment to flow cytometry as they have a range of forward and side scatter characteristics and spectral properties that can occlude and greatly outnumber stained leukocytes. Density gradient (Ficoll or Percoll) depletion of nucleated RBCs prior to flow cytometry is effective in removing most RBCs (Debuque and Godwin, 2015), but dual CD18/IB4 staining allows the clear identification of contaminating RBCs (Figure 1A). Axolotl myeloid cells can be identified with the CD18 (B2 integrin) surface antigen which forms a subunit for many granulocyte receptors. Isolectin B4 (IB4) also reacts with myeloid cells in multiple animal species (Sorokin and Hoyt, 1992; Zammit et al., 1993; Debuque and Godwin, 2015; Lai et al., 2017). B cells can be identified with the axolotl-specific Pan Ig marker (Tournefier et al., 1988a,b). In a large screen of anti-human antibodies, we identified a cross-reactive anti-CD2 monoclonal antibody that reacts with a subset of B cells (Figure 1A). This panel was used for FACS purification to isolate five major populations labeled A–E that were then profiled with downstream RT-PCR and cytological analysis (Figures 1A–D). Peripheral blood cell subset frequencies in the axolotl have monocyte/macrophage levels in a similar range to human, mouse, and rats. The T cell numbers are more consistent with human blood, while the B cell numbers are more consistent with rodent blood. Granulocyte counts are more consistent with the intermediate levels found in the blood of rats than the low levels in mice or high levels in humans (Figure 1B and Supplementary Figure 1).

Quantitative RT-PCR analysis using marker genes associated with distinct cell types showed high levels of purity in the five sorted populations (Figure 1C). The typical monocyte/macrophage receptor CSF1R (Rojo et al., 2019) is only enriched in population A. Granulocyte-associated genes (NE, MPO, and PRTN3) (Hirche et al., 2005) were significantly enriched in populations A and B (Figure 1C). This may be due to a small amount of granulocyte contamination in population A or could be co-expressed in both populations. The myeloid-specific genes ITGAM and PU.1 (Pahl et al., 1993) were both co-expressed in populations A and B (Figure 1C). The T cell-specific genes CD3 and TCRa (Xu et al., 2020) were enriched in population C as was the T-cell associated gene perforin. The B cell-specific genes IGHM and IG-lambda-Constant chain (Andre et al., 2000) were only enriched in population D and E. RAG-1 is a gene involved in T and B cell development and is downregulated during maturation (Durand et al., 2000). Some RAG-1 expression was detectable in population D and E, possibly indicating the presence of immature B cells circulating in the bloodstream (Figure 1C). The RT-PCR results were confirmed with Wright-Giemsa stained cytospin preparations of each population (Figure 1D). The cytospin preparations also demonstrate the purity of each sorted population. Population A has morphology consistent with monocyte/macrophages, population B is consistent with granulocytes (mostly neutrophils), and populations C–E have typical lymphocyte morphology with no visible contamination from myeloid cells (Figure 1D).

### Myeloid Cells Are the Major Circulating Leukocytes Recruited to Early Regenerating Wounds

**FIGURE 2:**
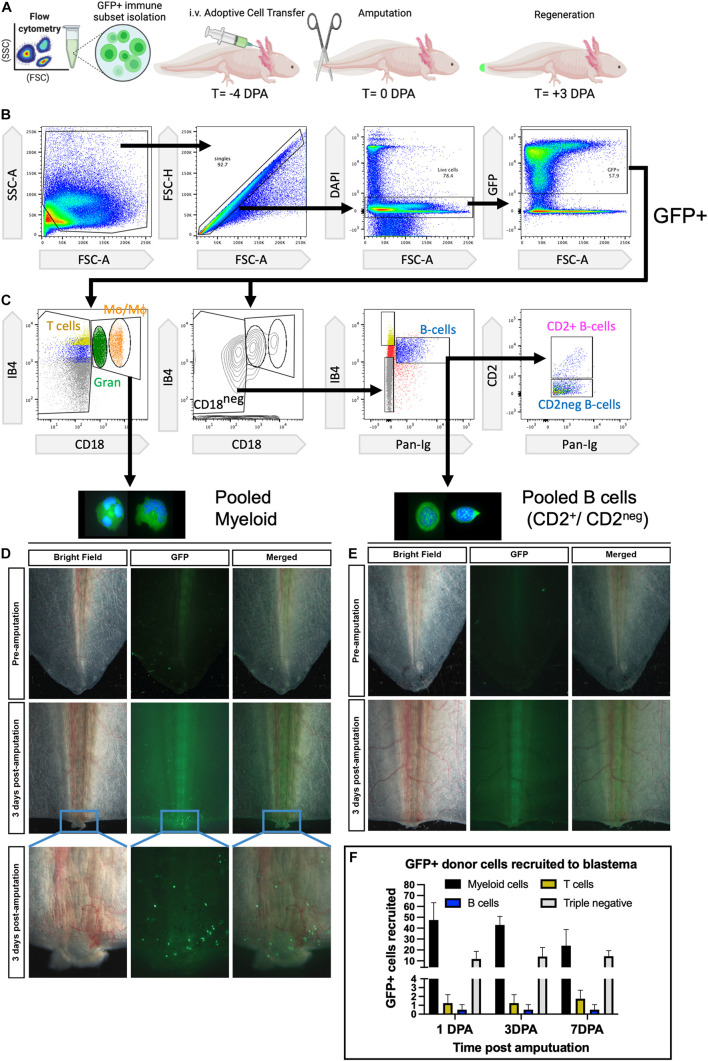
Myeloid cells are the major circulating leukocytes recruited to early wounds. **(A)** Experimental design to test peripheral blood recruitment to wounds. FACS isolation of peripheral blood leukocytes obtained from transgenic Tg:CAGGS:GFP (ubiquitous GFP + axolotls) and adoptive transfer of 5 × 10^4^ GFP^+^ donor cells before tail amputation and live cell imaging at 3 dpa. **(B,C)** FACS gating strategy for isolation of viable single cells and collection of myeloid cells and B lymphocytes. **(D)** Representative images show that myeloid cells are consistently detected in early 3-day wounds of amputated tails. **(E)** No detectable recruitment of GFP^+^ Pan-Ig + B cells is observed in 3-day wounds of amputated tails. *N* = 4 host recipients per transplant group. **(F)** Recruitment of major leukocyte cell types quantified at 1, 3, and 7 dpa. DPA, days post amputation.

Using the flow cytometry gating strategy we developed, we were able to isolate GFP^+^ myeloid and lymphoid B cells from peripheral blood and inject these into naïve hosts to profile the early time course of wounding for myeloid vs. lymphoid recruitment (Figures 2A–C). In the tail amputation model, we identified robust recruitment of myeloid (granulocyte and macrophage) GFP^+^ donor cells to regenerating wounds over the first 7 days, but no major recruitment of lymphoid GFP^+^ donor B cells (Figure 2F). To confirm that the lack of B cells was not due to a loss in B cell survival, we examined host liver and spleens at 15 days post adoptive transfer and confirmed a robust viable population of B cells recruited to host liver and spleens (Supplementary Figure 5). At 3 days post amputation (dpa), myeloid cells were robustly recruited to tail wounds and were detectable between 18 h and at least 7 dpa (Figure 2D and Supplementary Figure 2). Almost no donor-derived B cells could be detected between 18 h and 14 days (Figure 2E and Supplementary Figure 2). Both CD2^+^ and CD2^*neg*^ B cells exhibited similar phenotypes (Figure 2C and Supplementary Figure 2). CD18^+^ IB4^+^ macrophage adoptive transfers (without granulocytes) confirmed macrophage recruitment between 1 and 7 dpa. The CD18^*neg*^IB4^+^ putative T-cell population showed a small number of GFP^+^ donor cells arriving at 1 dpa and no major accumulation up to 7 dpa (Figure 2C and Supplementary Figure 2). Interestingly the triple-negative (CD18^*neg*^IB4^*neg*^PanIg^*neg*^) population that has yet to be identified did show some recruitment by 18 h post-amputation and was still visible up to 14 dpa (Figure 2C and Supplementary Figure 2). Taken together, myeloid cells and CD18^+^IB4^+^ macrophages are the dominant leukocyte recruited to regenerating wounds, which is consistent with their known pro-regenerative activities.

### Robust Homing of Liver-Derived GFP^+^ Cells to Amputated Limbs and Periphery Following GFP^+^ Organ Tissue Grafts Into Leucistic Hosts

**FIGURE 3:**
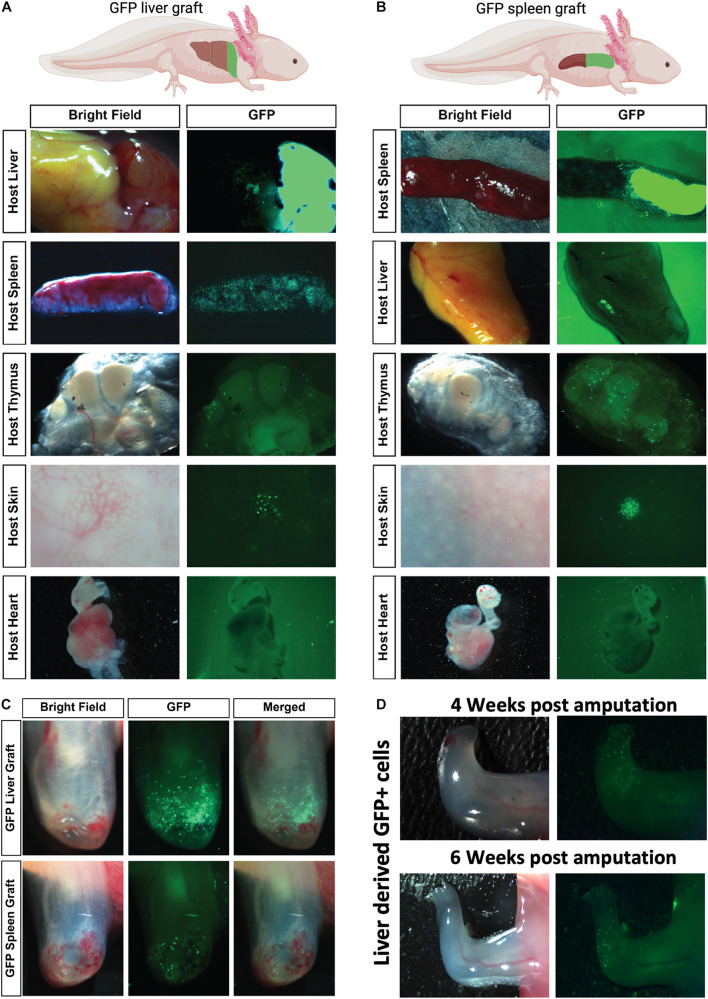
Robust homing of liver-derived homing GFP^+^ cells to amputated limbs and periphery following GFP^+^ organ tissue grafts into leucistic hosts. **(A,B)** Representative *ex vivo* imaging of host tissues 7 days post organ tissue graft. GFP^+^ liver grafted to host liver show that cells originating from this tissue have capacity to home in large numbers to the host liver, spleen but not the thymus, skin, or heart. GFP^+^ spleen grafted to host spleen show that GFP^+^ spleen cells can migrate in large numbers to the spleen and thymus and concentrated areas in the skin but show reduced migration to the liver and no migration to the heart. *N* = 4 host recipients. **(C)** Representative images of robust liver-derived GFP^+^ cell recruitment. Limbs were amputated 1 week after organ tissue graft and imaged to detect GFP^+^ cells migrating from graft to limbs 4 dpa. **(D)** Liver-derived GFP + cells still visible in regenerating limb 4 and 6 weeks post amputation (*N* = 4).

Both liver and spleen are thought of as the major contributors to adult hematopoiesis. Although the spleen is a major host for hematopoietic stem cells (HSCs) in the axolotl (Lopez et al., 2014), the exact site of adult myelopoiesis is still unknown. To test which organs are potentially responsible for acting as sites of myelopoiesis or reservoirs for mature myeloid cells prior to recruitment to the regenerating limb, we established an organ grafting model. It could be reasoned that myeloid-committed stem cells may need to reside inside a stem cell niche to be functional and undergo development and maturation before recruitment to peripheral sites. The grafting model is predicted to allow potential stem cells to maintain their niche and undergo normal development before testing their recruitment to the amputated limb. Taking advantage of the salamander’s ability to accept long-term allografts without rejection for 3–8 weeks (Tournefier et al., 1998; André et al., 2007), we resected the rostral tip of the host liver and replaced it with the equivalent region from a GFP^+^ donor. The grafts rapidly fused to the host tissue (Figure 3A and Supplementary Figure 3), which allowed us to test the potential recruitment of liver-derived cells to peripheral immunological sites or limb amputations (Figure 3A). Similarly, we were able to test the potential for spleen-derived myeloid cells by replacing the rostral half of the equivalent region with donor GFP^+^ spleen (Figure 3B). These grafting studies demonstrated that liver-derived GFP^+^ cells were robustly recruited to the spleen with very little if any migration to the thymic nodes or heart and minor recruitment to dermal vascular beds in the skin (Figure 3A). In contrast, splenic grafts showed robust recruitment of donor GFP^+^ cells to the thymic nodes and heavy accumulation localized in dermal nodes within the host skin (Figure 3B). Very little migration from the spleen grafts to the host liver was observed. After 1 week of donor-host engraftment, we performed limb amputations and examined the recruitment of donor GFP^+^ graft-derived cells to the amputated limb at 4 dpa (Figure 3C). These experiments identified robust donor GFP cell recruitment with GFP^+^ liver grafts but very weak recruitment to the amputated limb with GFP^+^ spleen grafts. Liver-derived GFP^+^ donor cells could be found in the regenerating limbs for up to 6 weeks (Figure 3D). These experiments suggest that liver is the major source of myeloid cells recruited to the regenerating limb.

### The Liver Is the Dominant Contributor of Myeloid Cells During Limb Regeneration

**FIGURE 4:**
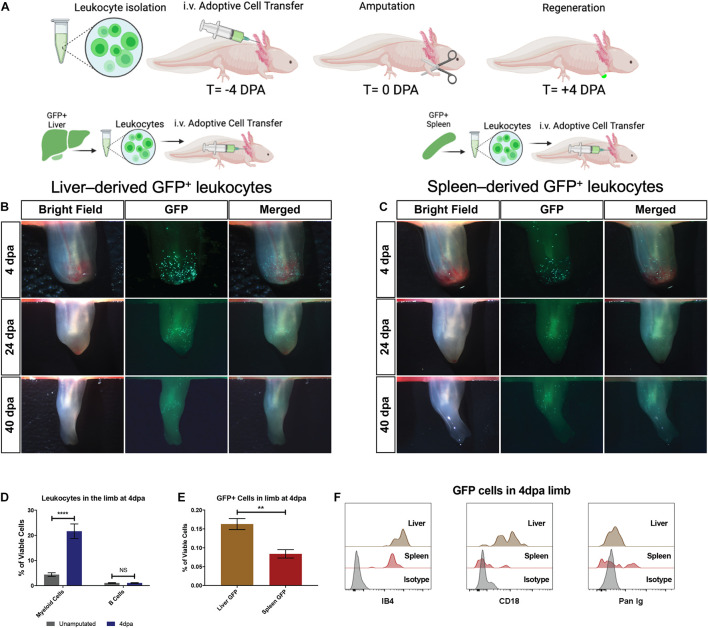
The liver is the dominant contributor of myeloid cells during limb regeneration. **(A)** Experimental strategy to identify cellular contributions from the liver and spleen following transplantation of 5 × 10^5^ live GFP^+^ cells. **(B,C)** Representative live imaging of the regenerating host limb at the wound healing (4 dpa), blastema outgrowth (24 dpa), and re-development (40 dpa) stages of limb regeneration. Liver-derived GFP^+^ cells are most abundant during the wound healing stage (4 dpa) and reduce in number throughout later stages of regeneration. Spleen derived GFP cells are recruited to the limb but qualitatively less in number compared to liver transplanted cells. *N* = 12 biological replicates. **(D)** Quantitation of myeloid cells and B cells in the limb at 4 dpa using CD18/IB4 and Pan-Ig staining, respectively. Bar graphs show mean ± SEM of four biological samples. *p*-Values obtained via one-way ANOVA with comparisons to the unamputated sample. ****p ≤ 0.0001. **(E)** Quantitation of viable GFP^+^ cells in the early regenerating limb following liver or spleen transplantation. Bar graphs show mean ± SEM of three biological samples. *p*-Values obtained via one-way ANOVA. **p ≤ 0.01. **(F)** Flow cytometry histograms of GFP^+^ cells in the limb testing for myeloid cell identity (CD18) in the second plot and B cell identity in third plot. T, time; DPA, days post amputation. ns, not significant.

To confirm our findings with GFP^+^ liver or spleen tissue grafts, we next tested the potential for dissociated GFP^+^ liver and spleen-derived single cells to migrate to amputated limbs (Figure 4A). This single-cell adoptive cell transfer (ACT) strategy used equal amounts of GFP^+^ cells from liver or spleen and from matched donors to quantify the homing potential for spleen-derived or liver-derived GFP^+^ donor cells. The recipients were full siblings to allow the closest possible match. Given that salamanders are extremely tolerant of grafts for up to 60 days (Tournefier et al., 1998; André et al., 2007) and lack the acute rejection responses that are present in mammals, it is reasoned that matching the donor tissue is the most important variable within this experimental design. The results supported the findings of the organ tissue grafts and demonstrated that liver-derived myeloid cells have greater homing potential for the amputated limb than splenic-derived donor cells in this model (Figures 4B,C,E). The majority of the GFP^+^ cells homing to the limb were found to be CD18^+^IB4^+^ myeloid cells with a flow cytometry profile (dual histogram peaks in both IB4 and CD18) that is consistent with a mixture of neutrophils and macrophages (Figures 4D–F).

### Identification of the Major Leukocyte Subsets in the Axolotl Liver and Spleen Throughout Its Lifespan

**FIGURE 5:**
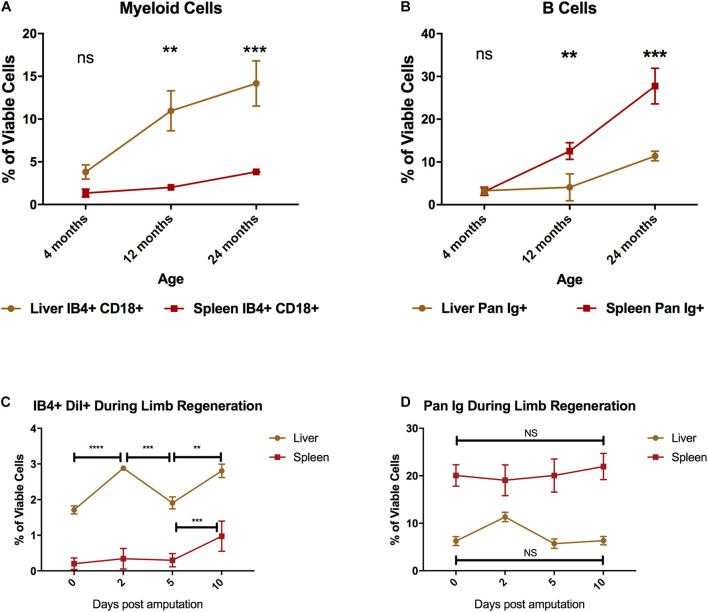
Liver and spleen identification of the major leukocyte subsets in the axolotl liver and spleen throughout its lifespan. **(A)** Line graph of percentage of myeloid cells (IB4^+^ CD18^+^) in the liver (brown) and spleen (red) prior to sexual maturity (4 months), sexual maturity (12 months), and thymic involution (24 months). **(B)** Line graph of percentage of B cells (Pan Ig^+^) in the liver (brown) and spleen (red). Graphs show mean ± SEM of three biological samples at each time point. Adjusted p-values obtained via 2-way ANOVA with multiple comparisons to the 4-month-old sample. ***p ≤ 0.01, ***p ≤ 0.001 **(C)** Line graph showing changes in mature phagocyte number (IB4^+^ DiI^+^ cells) measured by flow cytometry in the liver (brown line) and spleen (red line) over the course of early limb regeneration. **(D)** Line graph showing changes in B cell numbers measured by flow cytometry in the liver (brown) and spleen (red) over the course of early limb regeneration. Error bars represent mean ± SEM of three animals per group at each time point. Adjusted *p*-values obtained via one-way ANOVA with multiple comparisons within each group. **p ≤ 0.01, ***p ≤ 0.001, ****p ≤ 0.0001. ns, not significant.

Myeloid cells that have migrated to the sites of inflammation are eventually replaced via two mechanisms: hematopoiesis or by having tissue reservoirs that meet these demands by deploying cells from hematopoietic or extramedullary niches. The liver and spleen have been identified as the major hematopoietic tissues in the adult axolotl (Lopez et al., 2014). We thus chose these two tissues to evaluate their potential as myeloid cell reservoirs across key time-points of the axolotl life-span: juvenile (4 months), sexual maturity (12 months), and onset of thymic involution (24 months) (Durand et al., 2000; André et al., 2007). We then examined the changes in both myeloid cells and B cells in the liver and spleen of young, sexually mature, and older animals to attempt to identify major immunological changes in these representative myeloid/lymphoid subsets. We found that the liver maintained the production and/or housing of myeloid cells throughout the ages we tested and had a significant increase in myeloid cell signal over time. Flow cytometry analysis of these tissues at 4 months post fertilization showed that the liver contained more CD18^+^ IB4^+^ myeloid cells than the spleen (Figure 5A: 3.8% ± 0.83 vs. 1.34% ± 0.28). By 12 months, the liver contains an approximately fivefold greater portion of myeloid cells than the spleen (Figure 5A). We found no significant changes in the spleen with age. Conversely, we found no significant accumulation of B cells in the liver, but B cell numbers steadily increased in the spleen with the age of the animal (Figure 5B). The spleen was found to be the primary site where B cells resided in all tested time points, and they comprise nearly one third of the whole spleen tissue by 24 months of age (Figure 5B).

To examine potential changes in macrophage or B cell populations with the liver or spleen that could be consistent with a response to limb amputation, we set up an experiment to profile these organs over the first 10 days post amputation (dpa). Phagocytic myeloid cells are critical during the early phases of regeneration and so we used a functional marker for phagocytic macrophages (uptake of fluorescent DiI liposomes (Debuque and Godwin, 2015)) that allows liver profiling. Using this labeling technique, we examined the liver and spleen at 2, 5, and 10 dpa to see if there was any disturbance in the liver myeloid phagocyte population or PanIg^+^ B cell numbers. After amputation, liver myeloid phagocyte numbers significantly fluctuated throughout 2–10 dpa, whereas in the spleen myeloid phagocyte numbers only significantly changed between 5 and 10 dpa (Figure 5C). B cell numbers did not significantly change in either liver or spleen tissue during the first 10 dpa (Figure 5D). The triple-negative (CD18^*neg*^IB4^*neg*^PanIg^*neg*^) as well as the putative T cell populations (CD18^*neg*^IB4^+^PanIg^*neg*^) appear relatively stable over 24 months in the liver and spleen (Supplementary Figure 4). Taken together, the change in liver myeloid cell numbers within the spleen in response to amputation are consistent with the liver being the major reservoir for myeloid cells that are recruited to amputated limbs.

### GFP^+^ Cell Homing in Serial Transplants Reveals Preference for Organ of Origin That Is Lost in Liver-Derived Cells That Have Been Educated in the Spleen

**FIGURE 6:**
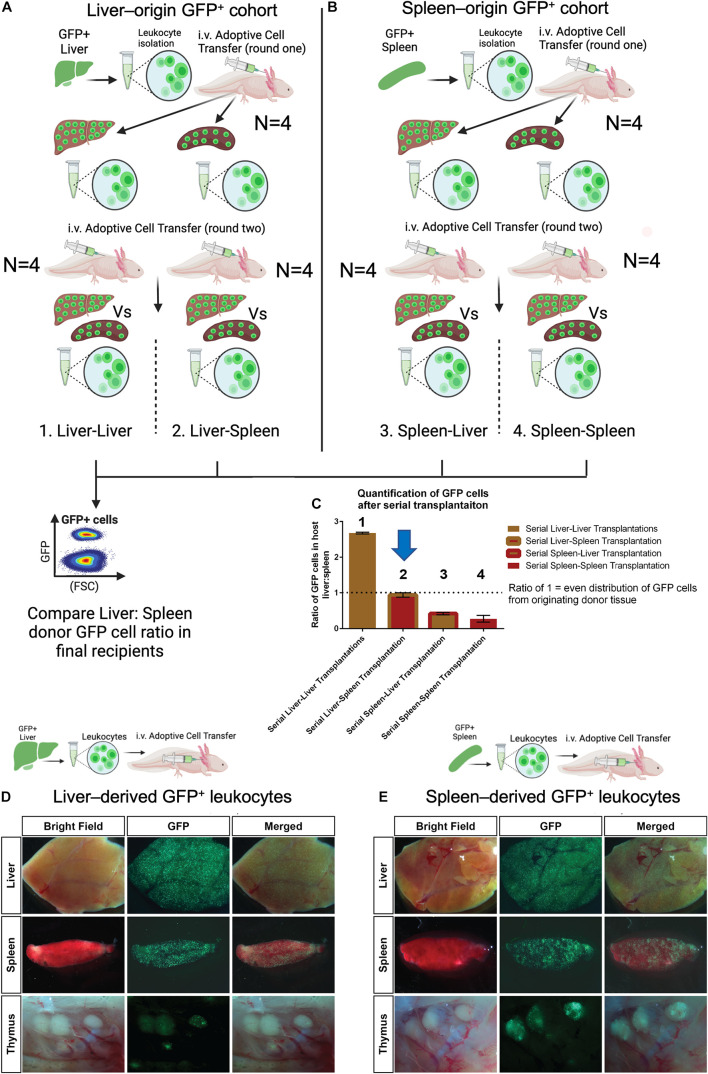
GFP + cell homing in serial transplants reveals preference for organ of origin that is lost in liver-derived cells that have been educated in the spleen. **(A)** Experimental strategy displayed as a cartoon depicting serial liver and spleen transplantation to quantify GFP cell homing bias. Liver-derived GFP^+^ cells from four individual animals were adoptively transferred into four separate non-GFP hosts (round one transfer). Seven days post transfer GFP^+^ cells were collected from livers and spleens in parallel (i.e., four liver-liver and four liver-spleen) These parallel GFP^+^ cell preparations were then adoptively transferred into one naïve host recipient per cell preparation (round 2 transfer). After another 7 days, both liver and spleens were isolated and profiled as paired samples (i.e., one liver and one spleen per host) by flow cytometry. **(B)** Spleen-derived GFP^+^ cells were tested as described for **(A)** using spleens from four GFP + animals as original donor tissue. (i.e., four spleens −>4 spleen-spleen + 4 spleen-liver transfers). **(C)** Flow cytometry cell counting of terminal organs containing GFP^+^ cells from serially transplanted organs. The ratio between terminal livers and spleens was quantified per recipient with a value of 1 indicating an equal preference of GFP^+^ cells for liver or spleen from round 2 adoptive transfers. Values higher than 1 indicate a preference for homing to host liver and values lower than 1 indicating a preference for homing to host spleen tissue. Only liver-derived GFP^+^ cells isolated from host spleen (condition 2, liver-spleen, indicated with blue arrow) show education that allows equal migration to liver/spleen from round two adoptive transfers. Bar graph showing the ratio of GFP cells in the liver and spleen from serial transplant experiments. Error bars represent mean ± SEM of four biological samples. **(D,E)** Intravenously transplanted GFP^+^ cells from the liver and spleen display differential leukocyte trafficking to host lymphoid organs with a preference for the tissue of origin. Spleen-derived GFP^+^ cells also show robust homing to thymic nodes. Representative *ex vivo* images of host lymphoid organs four days after transplantation of 1–5 × 10^5^ liver or spleen leukocytes. *N* = 6 host recipients in each transplant group.

In mice, immature myeloid cells can traffic to the spleen to be educated and finish their development before responding to infection or injury in the periphery (Swirski et al., 2009; O’Neill et al., 2011; Wu et al., 2018). Since the spleen had a delayed myeloid cell response relative to the liver (Figure 5C), we wondered about the potential for liver-derived myeloid cells to migrate to the spleen to acquire new functions possibly required for regeneration. We established an assay using a serial transplant model of organ-specific GFP^+^ adoptive cell transplants to examine inter-organ trafficking and potential homing biases (Figure 6). In round one of this assay, GFP + donor cell transplantations were performed with equal numbers of either liver-origin (*N* = 4) or spleen-origin (*N* = 4) donor cells, delivered to alternative recipient (GFP^–^) cohorts (*N* = 4 + 4) (Figure 6A). After 1 week, liver and spleens were isolated and dissociated in parallel from each cohort. GFP^+^ cells obtained from each organ were re-injected into the blood in equal cell numbers (Round 2) into new (GFP^–^) terminal recipients (*N* = 4 + 4 + 4 + 4). After another week, both spleens and livers were collected as paired samples from each of the 16 animals. To assess the effect of cross organ exposure (education) on liver-origin or spleen-origin GFP cells, we used flow cytometry to count GFP^+^ cells in the terminal recipients of paired liver and spleen tissue. We then calculated the ratio of GFP cells between liver and spleen per recipient animal where a 1:1 ratio would reflect that the GFP^+^ cell mixture that was adoptively transferred had an equal chance of trafficking to either the liver or spleen. Those animals with ratios higher than 1 would reflect a GFP^+^ donor cell population with a preference for liver. Those with a ratio lower than 1 represent donor GFP^+^ cells with a preference to home to spleens. The results (Figure 6) indicated that those GFP^+^ cells that have only been exposed to liver prefer the liver, and those that have only been exposed to spleen preferentially home to the spleen. Strikingly, those liver derived GFP^+^ cells that were recovered from spleens lost their preference for liver-specific homing, whereas, splenic derived GFP^+^ donor cells recovered from livers maintained their preference. These experiments indicate that liver-derived GFP^+^ cells and spleen-derived GFP^+^ cells are not equivalent, thus indicating that homing may be one directional from liver to spleen with splenic education potentially expanding cell functions. Finally, we confirmed that the primary migration of dissociated GFP^+^ cells in this model is functionally equivalent to the homing preferences obtained in the organ-transplant model. Although the dissociated cell transplant model shows reduced GFP^+^ cell migration relative to the organ transplant model (Figure 3), it has the major advantage that equalized cell inputs allow sensitive quantification.

### The Spleen Is Not Required for Liver-Derived Myeloid Cell Trafficking to the Limb or for Limb Outgrowth

**FIGURE 7:**
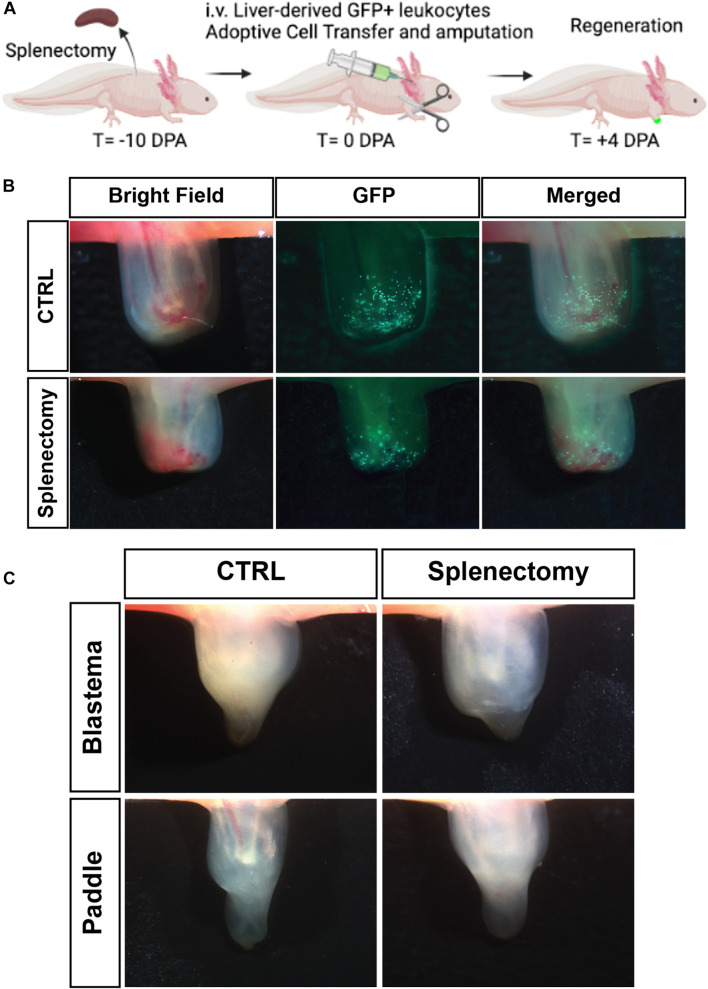
The spleen is not required for liver-derived myeloid cell trafficking to the limb or for limb outgrowth. **(A)** Experimental timeline to study cellular contributions from the liver following splenectomy. **(B)** Live imaging of 4 dpa limb. GFP cells in the limb following 5 × 10^5^ GFP liver cell transplant in non-splenectomized control cohort (Top row). GFP cells in the limb following 5 × 10^5^ GFP liver transplant in splenectomized control cohort (Bottom row). (*N* = 4 per group) **(C)** Live image of regenerating limbs with spleen (pictured left) and without spleen following splenectomy (pictured right). Critical regeneration stages shown. All animals regenerated their limbs in both groups. *N* = 8 animals per experimental group.

Having shown that there is potential for liver-derived cells to traffic to the spleen and alter the homing bias, we wanted to test the requirement for spleen in adult axolotl limb regeneration. By performing a splenectomy combined with adoptive cell transfer of liver-derived leukocytes from a GFP^+^ donor on white leucistic (d/d) animals (Figure 7A), we were able to confirm that the spleen is not required for liver-derived leukocyte cells to traffic to the amputated limb. We then wanted to test the requirement for the spleen in functional limb regeneration. Experiments where the spleen was removed prior to limb amputation confirmed that limb regeneration can occur in the absence of a spleen (Figure 7B) and that this regeneration occurs in a similar timeframe to reach the blastema stage, paddle stage, and full regeneration (Figure 7C). Taken together, this work strongly suggests that the liver is the primary organ responsible for the supply of myeloid cells participating in salamander limb regeneration.

## Discussion

The immune system is now appreciated to play a critical role in shaping the outcome of tissue repair in various animal models and tissue contexts (Godwin et al., 2017b; Mescher et al., 2017). In the salamander, the recruitment of macrophages to the amputation plane is an essential step in successful limb and heart regeneration (Godwin et al., 2013, 2017a). In mammalian wound healing, the origin and phenotype of recruited macrophages play a critical role in the regulation of scar formation and repair quality (Wynn and Vannella, 2016). Repair quality can also be shaped by the presence of other non-macrophage immune cells within the wound (Julier et al., 2017). In the salamander, there is lack of basic knowledge in the types of immune cells recruited to the regenerating wound and the hematopoietic origin of the cells that are recruited. To address this knowledge gap, this report provides a broad assessment on the origin and trafficking routes of leukocytes recruited to regenerating tissue in salamanders. We successfully developed a flow cytometry toolkit that efficiently identifies major leukocyte subsets in blood, organs, and regenerative tissues that was validated with downstream molecular and cytological analysis. Coupling of this toolkit, with adoptive cell transfers and organ tissue grafting in parallel, we identified that myeloid cells are the major leukocyte subtype recruited to regenerating wounds. Furthermore, this work revealed the adult liver as the primary source of myeloid cells recruited to regenerating wounds. Although surgical removal of the liver with survival is not possible, the removal of the spleen demonstrated that the spleen was unnecessary for myeloid cell recruitment to the regenerating limb and was also dispensable for regeneration. Given the robust recruitment of liver-derived macrophages to the amputated limb, in the absence of a spleen, the liver is the most likely primary source of pro-regenerative macrophages.

The frequency of each leukocyte population in healthy adult animals can vary widely by species and collection method. It is generally agreed that human blood is rich in granulocytes (50–70% granulocytes and 30–50% lymphocytes) with lower lymphocyte counts than rodents (5–25% granulocytes and 75–90% lymphocytes) (Mestas and Hughes, 2004). Interestingly, huge variation is observed between mice and rats in both lymphocyte and granulocyte cell frequencies (Supplementary Figure 1). Rats have unusually high T-cell counts compared to mice and humans, but all rodents have high B cell numbers (Supplementary Figure 1). The profiling of axolotl peripheral leukocytes revealed a profile with a monocyte/macrophage frequency in a range that is similar to humans and rodents. Despite known deficiencies in adaptive immunity (Cohen, 1971; Salvadori and Tournefier, 1996; Cotter et al., 2008), axolotl blood appears to have T-cell numbers that are equivalent to human and mouse blood, with B cell frequencies closer to rodents than humans. In terms of granulocyte counts, axolotl blood falls in a similar range as rats and humans, whereas mice seem to have an unusually low number of circulating granulocytes. The axolotl blood cell frequency analysis comes with a major caveat that 16–25% of the circulating leukocytes could not be identified by the staining panel that we used and that some of these cells may fall into those T cell, B cell, and granulocyte categories. It should also be noted that the existence and frequency of circulating NK, NKT, DC, thrombocytes, immature leukocytes, and circulating stem cells is yet to be determined. Taken together, adult axolotl blood appears to have cell frequencies that are intermediate between mouse and human peripheral blood frequencies.

Whilst several non-mammalian model organisms are utilized in regenerative biology, amphibians such as anurans (frogs) and urodeles (salamanders) offer a unique perspective to study the contributions of the innate immune system as they share a majority of the key immune tissues present in mammals (Ciau-Uitz et al., 2006; Demircan et al., 2016). The notable exception is bone marrow and its presence, or lack thereof, in many anuran amphibians and most urodele amphibians, excluding Plethodontidae (lungless salamanders) (Curtis et al., 1979). When no bone marrow is present, consensus view equates adult amphibian hematopoiesis to that of the developing avian and mammalian blood system, a period during which both animals retain high regenerative capacities (Mescher and Neff, 2005; Allender and Fry, 2008; Coleman, 2008; Larson et al., 2010). In these organisms, definitive hematopoiesis of hematopoietic stem cells (HSCs) originates in the aorta-gonad mesonephros (AGM), which expands and seeds the fetal liver, spleen, then thymus (Dzierzak and Speck, 2008). Studies have shown that unlike mammals, the axolotl does not form or utilize bone marrow as the primary tissue for definitive hematopoiesis following birth (Brunst, 1958; Durand et al., 2000; Golub et al., 2004; Lopez et al., 2014). Anuran amphibians similarly do not use bone marrow as the primary site for blood production. However, their propensity for scar-free repair and limb regeneration progressively declines following the onset of metamorphosis, which coincides with the maturation of its adaptive immune system (Mescher and Neff, 2006; Godwin and Rosenthal, 2014; Mescher et al., 2017). In contrast, salamanders retain regenerative proficiency following metamorphosis and are regarded as immunodeficient. This is characterized by a weak humoral response to soluble antigens, slow cytotoxic immune responses, and high vulnerability to viral infections due to defective T cell proliferation (Cohen, 1971; Salvadori and Tournefier, 1996; Cotter et al., 2008).

In addition to functioning as sites for hematopoiesis, our results indicate that the liver and spleen also serve as reservoirs for myeloid cells and B cells. This is in line with historical histological findings in several salamander species, which observed granulocytes primarily residing in the sub-capsular zone of the liver and lymphocytes predominantly inhabiting the white pulp regions of the spleen (Rowley and Ratcliffe, 1988; Tournefier et al., 1988b; Fini and Sicard, 2010). During mammalian development, the major site of hematopoiesis transitions from the fetal liver to the spleen and bone marrow (Sheng et al., 2015). In adult life, HSCs dynamically change their location and phenotypes, shifting from quiescent and stationary cells anchored in the bone marrow to cycling and motile cells entering circulation (Parkman and Weinberg, 2014). These changes are driven by stress signals. Bidirectional migrations to and from the bone marrow are active processes that form the basis for HSC transplantation protocols. In addition, fetal liver-derived HSCs are primed to seed the splenic tissue niche from which HSCs will be partially maintained in adult life. Splenic monocyte reserves are known to be mobilized to liver tissues when inflammation occurs (i.e., infection or injury) (Wynn et al., 2014). However, the potential for reverse migration from spleen to liver under homeostatic conditions in mammals has not been adequately evaluated. Given the dynamic regulation of HSCs in mammals, it is therefore not entirely surprising that leukocytes exhibit bidirectional trafficking and a bias toward their tissue of origin. It is notable that salamander liver cells captured from host spleens lose their bias toward liver and exhibit new migration potential. This finding suggests that there is an altered phenotype potentially involving an educational process induced by the splenic microenvironment. Despite this altered functional readout, splenic modification of liver-derived leukocyte phenotype is not a requirement for effective limb regeneration.

A recent study conducted by Lopez et al. (2014) has pointed to the adult axolotl spleen as the primary tissue for leukocyte deployment following hematopoiesis into peripheral tissues, such as the skin. These results were obtained by grafting a GFP labeled cephalic portion of one embryo to the RFP or wild-type caudal segment of another. These experiments did not characterize leukocyte identity or assess leukocyte contributions during regeneration. In contrast, our dual-model approach utilizing tissue grafting and cell transplantation of age-matched GFP donors into leucistic hosts confirmed the liver as the principal organ for myeloid cell deployment into the early regenerating limb. While the study by Lopez et al. (2014) provides critical information on the contribution of splenic hematopoietic progenitors that give rise to skin resident leukocytes in adults, our approach has revealed that the major source of leukocytes recruited to sites of regeneration are myeloid cells that originate in the liver. Whilst our study tracked liver-derived GFP myeloid cells migrating into the amputated limb, it is not yet clear what cell type accounts for the numerous cells observed in the skin that are of spleen origin. Clarity on this matter will be resolved with the development of transgenic tools specific to diverse immune cell lineages.

Regeneration of the mouse heart is observed during a narrow temporal window during development and is gradually lost between 1 and 7 days of postnatal life (Porrello et al., 2011). This loss of regenerative potential is correlated with the switch from dependence on fetal liver-derived hematopoiesis to bone marrow derived hematopoiesis. Importantly, this process has been shown to be critically dependent on macrophages, and considerable collateral evidence is emerging that fetal liver-derived macrophages exert a higher potential for tissue repair (Dey et al., 2014). It is possible that salamanders maintain a hematopoietic system in adult life that is analogous to the fetal liver-dominated regeneration-competent stage of mouse development. Our findings support a model where the emergence of a bone marrow-derived hematopoietic immune system may be inhibitory for regeneration.

Sustained myeloid cell recruitment into the site of injury is a conserved process between salamander and mammalian tissues. Blockade of this process in the axolotl results in the formation of fibrotic tissue similar to that induced in the injured mouse heart characterized by the abnormal appearance of myofibroblasts and an irregular build-up of thick collagen I and IV fibers in the place of thin collagen III (Aurora et al., 2014; Godwin et al., 2017a). Our findings implicate the axolotl liver as the niche in which these macrophages are educated prior to deployment into the limb where they act as positive regulators of regeneration. An essential element of leukocyte biology is the definition of progenitors and precursors since this underpins the formation of cell lineages with distinct functions. Further examination into how this niche responds to systemic injury signals to instruct the appropriate myeloid cell phenotype release into regenerating tissues is warranted. Juxtaposing these basic mechanisms to analogous models that display ranging competencies for tissue regeneration will outline the necessary elements required for adaptation toward clinical settings.

## Materials and Methods

### Ethics Statement

All scientific procedures involving animals were undertaken in line with Animal Ethics Committee guidelines for Monash University or the MDI Biological laboratory.

### Animal Husbandry, Procedures, and Live Imaging

*Ambystoma mexicanum* (Mexican axolotl) animals (wild-type, d/d leucistic “white hosts” and Tg:CAGGS:GFP) were obtained from the Ambystoma Genetic Stock Centre (AGSC), Lexington, KY, United States, and captive bred. Animals were individually housed in carbon-filtered tap water tanks on a 12-h light, 12-h dark cycle. Juvenile and adult animals were used for all experiments and were between 4 and 24 months old. Prior to animal surgeries (blood collection, amputation, tissue transplantation, and live imaging), animals were anesthetized using 0.1% ethyl-3-aminobenzoate methanesulfonate salt (MS-222; Sigma-Aldrich, St. Louis, MO, United States). Forelimb and tail amputation procedures were carried out as previously described (Godwin et al., 2013; Debuque and Godwin, 2015). For cell transplantation experiments, 1–5 × 10^5^ cells were delivered intravenously into the dorsal side of the gills by adapting previous intravenous delivery methods (Debuque and Godwin, 2015).

Cell transplantation experiments were carried out in 5–7-month-old age-matched animals (snout to tail length of 8–12 cm). Liver and spleen grafts were performed on 9–12-month-old animals (snout to tail length of 15–20 cm) where 25% of the rostral side of the host organ was resected and then equal volume of donor tissue was grafted onto the host. For live imaging procedures, harvested organs or anesthetized animals were imaged using a Zeiss Lumar V12 fluorescent stereo microscope. Spleens were removed via keyhole surgery. The remaining vessels were cauterized to prevent any blood loss and the incision into the skin was sutured. The control cohorts for these experiments underwent sham surgery.

### Isolation of Single Cell Suspensions From Peripheral Blood, Liver, and Spleen

Peripheral blood was isolated as previously described (Debuque and Godwin, 2015) with minor adaptations (using animals’ snout to tail length of around 20 cm and at least 12 months of age). Briefly, peripheral blood was collected from the gills facing the ventral side in anesthetized animals using a 25G SURFLO^®^ winged infusion set (Terumo Medical Corporation, Somerset, NJ, United States) and was dispensed into 50-ml tubes containing ice-cold 0.7X HBSS-5 mM EDTA. Cells were then centrifuged at 200 × *g* at 4°C for 20 min (with no decelerating brakes) to remove platelets. Contaminating red blood cells were then removed by layering the cell suspension onto Ficoll Paque Plus (GE Healthcare, Waukesha, WI, United States) and centrifuging the sample for 5 min at 400 × *g* at room temperature (no decelerating brakes). All liquid phases except the red blood cell phase were collected and washed. Liver and spleens were harvested from euthanized animals and briefly rinsed in ice-cold 1X HBSS/5 mM EDTA and placed onto a 10-mm tissue culture dish. Tissues were then processed with surgical scissors and minced into fine chunks of approximately 25 mm in cubic size then processed through a 70-μM cell-strainer with the aid of a 3-mL syringe plunger. Cells were then centrifuged twice at 200 × *g* at 4°C for 20 min with no decelerating brakes to remove tissue debris, and their numbers were counted using the Countess Automated Cell Counter (Thermo Fisher Scientific, Carlsbad, CA, United States).

### Flow Cytometry and FACS

Single cell suspensions were blocked with ice-cold 1 × HBSS/5 mM EDTA/1X DNase1/2% goat serum for at least 20 min on ice as previously described (Debuque and Godwin, 2015). Primary antibodies were then added at final dilution and incubated for at least 1 h on ice shielded from light. Primary antibodies were washed, and if necessary, cells were then incubated with their corresponding secondary antibody for 30 min on ice shielded from light. Cells were then washed and transferred into FACS tubes for flow cytometry analysis or FACS isolation. Cell viability for flow cytometry experiments was assessed with Ghost Dye^TM^ Red 780 (Tonbo Biosciences, San Diego, CA, United States) following manufacturer’s instructions. Cell viability for FACS experiments was assessed using DAPI. FACS was performed using either the BD symphony A6 or BD Influx Cell sorters. Flow cytometry quantification was performed on LSRII Flow Cytometers (BD Biosciences, San Jose, CA, United States). Compensation of fluorescence spectral overlap was used with UltraComp eBeads (eBioscience, San Diego, CA, United States) according to manufacturer’s instructions. FCS 3.0 files generated by flow cytometry were analyzed using FlowJo Software v10. For list of antibodies used in these experiments and their working dilutions, see Supplementary Table 1.

### Giemsa–Wright Stain

Cytospins and staining was performed as described in Debuque and Godwin (2015). Briefly, following FACS isolation, cells were transferred onto poly-L-lysine slides utilizing a cytospin centrifuge and then fixed in 4% PFA. Working dilution May–Grunwald stain was applied to cells for 5 min and then washed after which working dilution Giemsa stain was applied for 15 min. Following staining, slides were then rinsed multiple times with distilled water, air-dried, and preserved in DEPEX and imaged under a light microscope.

### cDNA Synthesis and RT-qPCR

Cell samples were collected into TRIzol^®^ reagents (Thermo Fisher Scientific). RNA was purified using Direct-Zol^TM^ RNA MiniPrep (Zymo Research, Irvine, CA, United States) according to manufacturer’s instructions. RNA quality was assessed by spectrophotometry using a NanoDrop ND-1000 (NanoDrop). Reverse transcription was performed using SuperScript^®^ VILO^TM^ cDNA synthesis kit (Thermo Fisher Scientific). Quantitative polymerase chain reaction (qPCR) assays were performed using LightCycler^®^ 480 SYBR green (Roche, Indianapolis, IN, United States). Gene expression levels were calculated using the 2^–ΔΔCt^ method. Sample gene expression was normalized to the geometric mean of three housekeeping genes and either expressed as relative fold change or log_2_ fold change. Primer sequences used in qPCR assays are listed in Supplementary Table 2.

### Statistical Analysis

Statistical analyses were performed using the software Prism 9 (GraphPad Software, San Diego, CA, United States). Data are always shown as mean values ± SEM. Analyses of significant differences between means were performed using unpaired Student’s *t*-test, one-way ANOVA for cell counts, or a two-way ANOVA with Turkey’s multiple comparisons test for RT-PCR comparisons between groups as indicated in figure legends.

## Data Availability Statement

The raw data supporting the conclusions of this article will be made available by the authors, without undue reservation.

## Ethics Statement

The animal study was reviewed and approved by Monash University Animal Use and Care Committee MDI Biological Laboratory IACUC.

## Author Contributions

RD and JG conceived and designed the experiments, and wrote the manuscript. RD, AH, and GJ performed the experiments and analyzed the data. NR provided funding, infrastructure, and editorial support for the manuscript. All authors contributed to the article and approved the submitted version.

## Conflict of Interest

The authors declare that the research was conducted in the absence of any commercial or financial relationships that could be construed as a potential conflict of interest.

## Publisher’s Note

All claims expressed in this article are solely those of the authors and do not necessarily represent those of their affiliated organizations, or those of the publisher, the editors and the reviewers. Any product that may be evaluated in this article, or claim that may be made by its manufacturer, is not guaranteed or endorsed by the publisher.
