# Supplementary material for: Identification of the Adult Hematopoietic Liver as the Primary Reservoir for the Recruitment of Pro-regenerative Macrophages Required for Salamander Limb Regeneration
Source: Front Cell Dev Biol. 2021 Sep 22;9:750587. doi: 10.3389/fcell.2021.750587 (PMC8456783; doi:10.3389/fcell.2021.750587)
Supplement: Supplementary file 1 [file Data_Sheet_1.docx]

**Identification of the adult hematopoietic liver as the primary reservoir for the recruitment of pro-regenerative macrophages required for salamander limb regeneration.**

**Ryan J Debuque^1^, Andrew J Hart^3^, Gabriela H Johnson^3^, Nadia A Rosenthal^1,2^ and James W Godwin^,1,2,3,*^.**

Supplementary Material

**Fig S1, (A).**
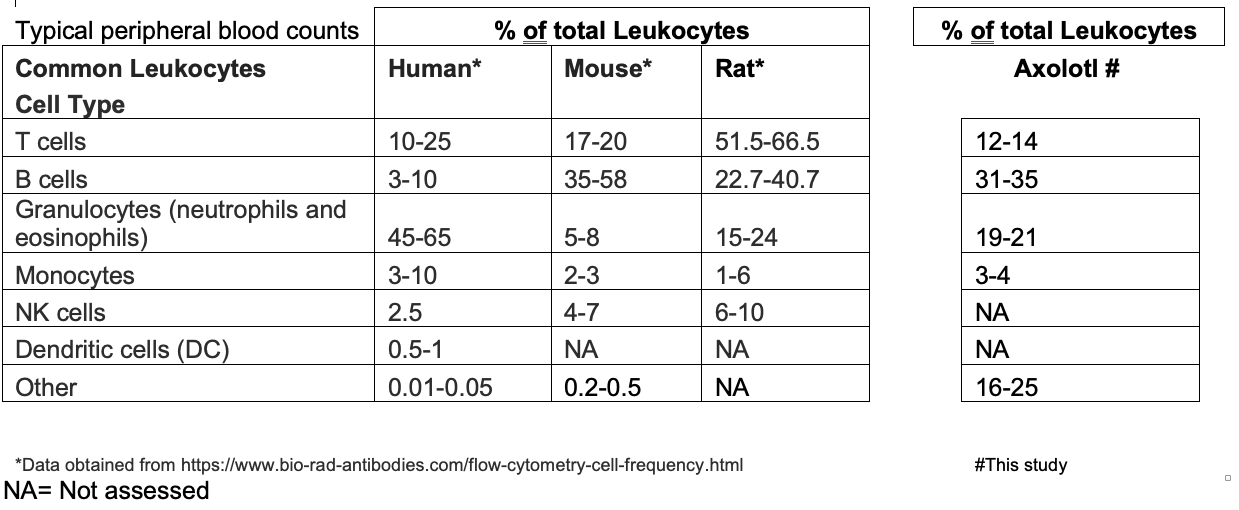


**Fig S1, (B)**

**Fig. S1. Typical non-RBC leukocyte counts found in axolotl compared with those reported for human, mouse and rat. (A)** Range of leukocyte frequency for major cell subsets reported at Bio-Rad.com compared with this study. **(B)** Average cell frequencies from (A) plotted as “parts of whole” using Prism Graphpad as a visual aid.

**
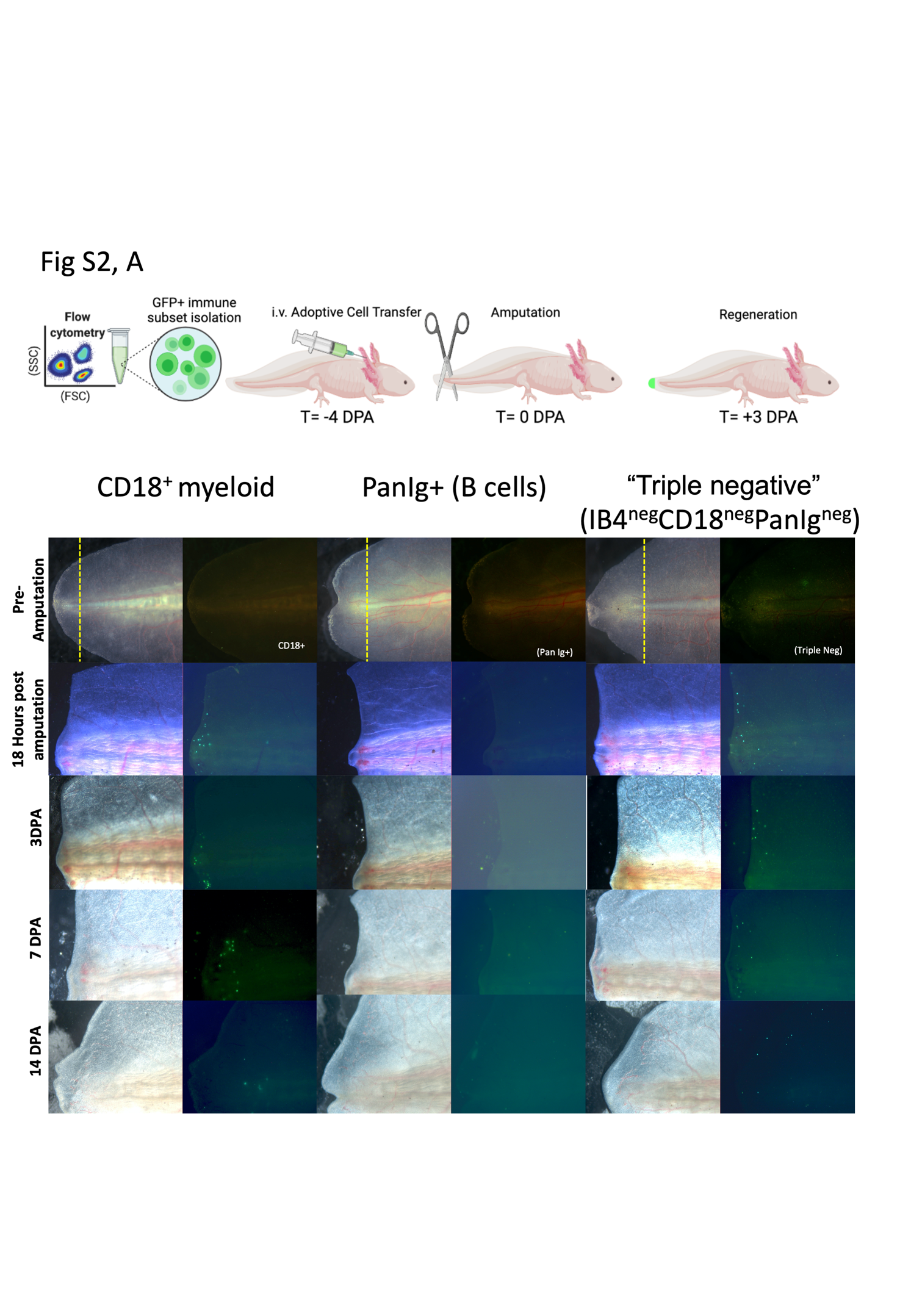
**

**Fig. S2, (A)** Representative adoptive transfer experiments testing the potential for FACS sorted populations to traffic to amputated tails. Time course of GFP^+^ cell recruitment at 18 hours, 3, 7 and 14-days post amputation comparing myeloid cells (CD18^+^), B cells (PanIg^+^) and triple negative (IB4^neg^CD18^neg^PanIg^neg^) donor cells to the regenerating tail (N=5).


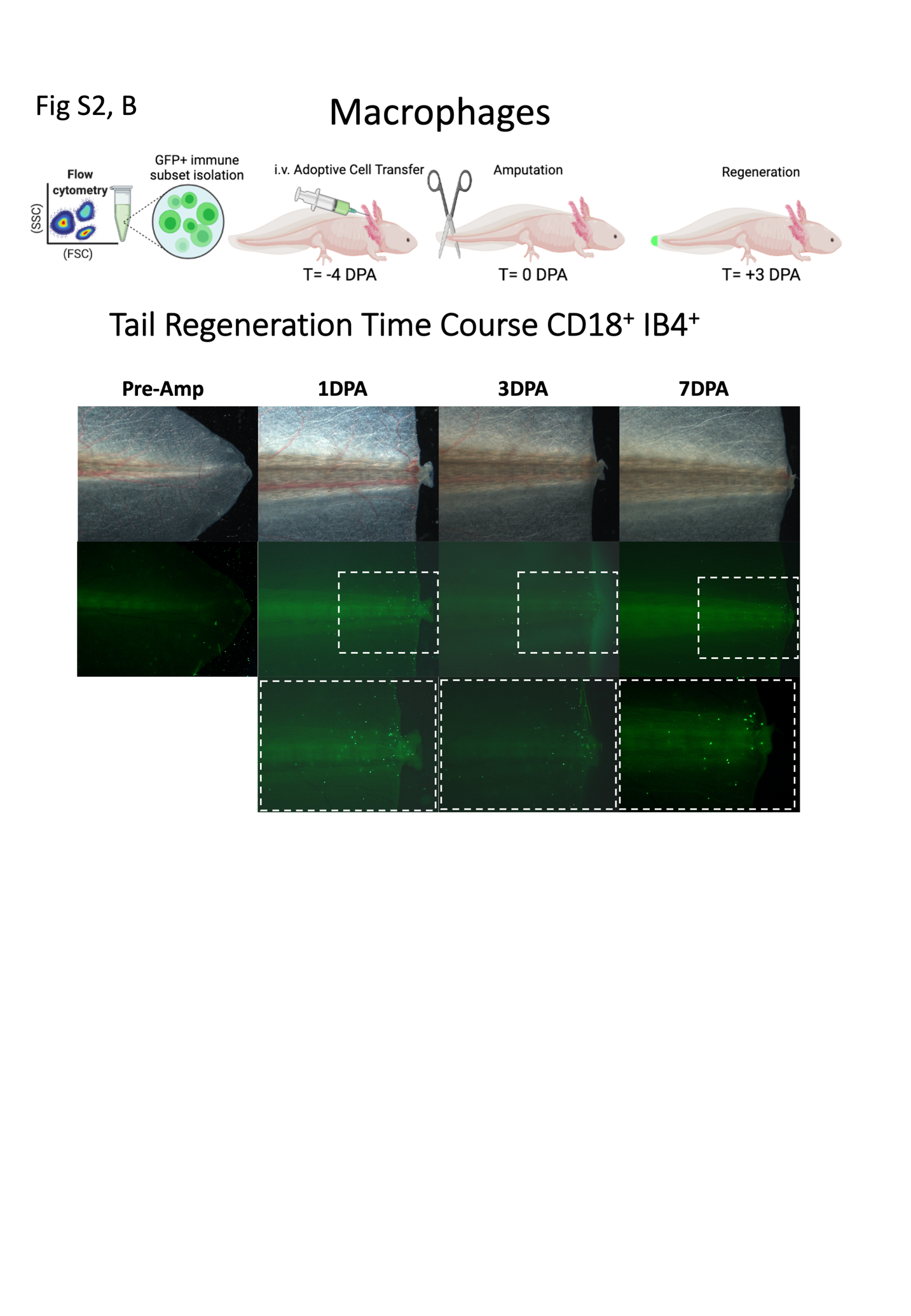


**Fig S2, (B)** Representative adoptive transfer experiments testing the potential for FACS sorted populations to traffic to amputated tails.Time course of monocytes/macrophage (IB4^high^CD18^+^) GFP^+^ donor cell recruitment at 1, 3 and 7 dpa days to the regenerating tail (N=6).


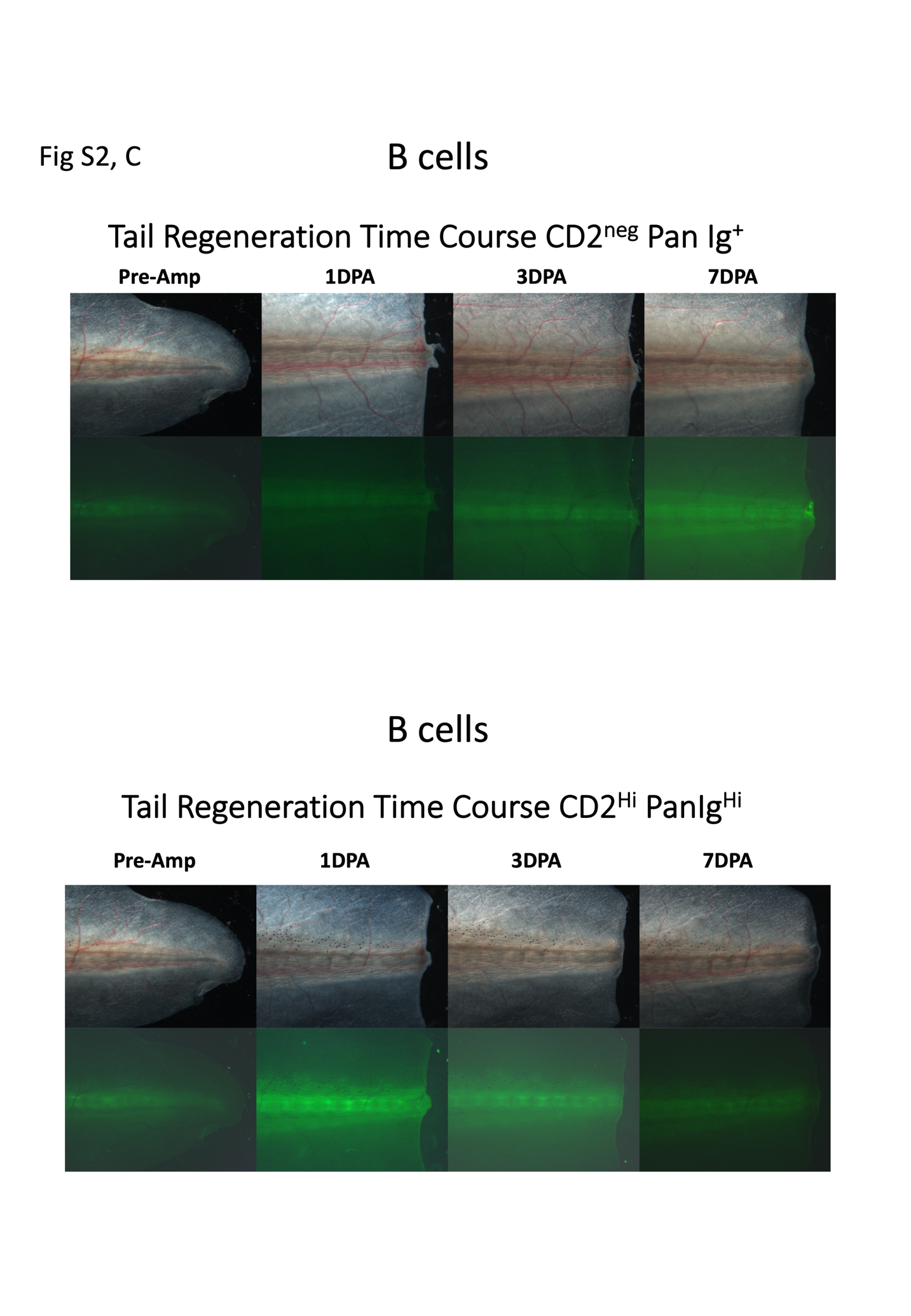


**Fig S2, (C)** Representative adoptive transfer experiments testing the potential for FACS sorted populations to traffic to amputated tails.Time course comparing CD2^neg^ B cells (PanIg^+^) and CD2^neg^ B cells (PanIg^+^) GFP^+^ cell recruitment at 1, 3 and 7 dpa days (N= 4 + 2).

**
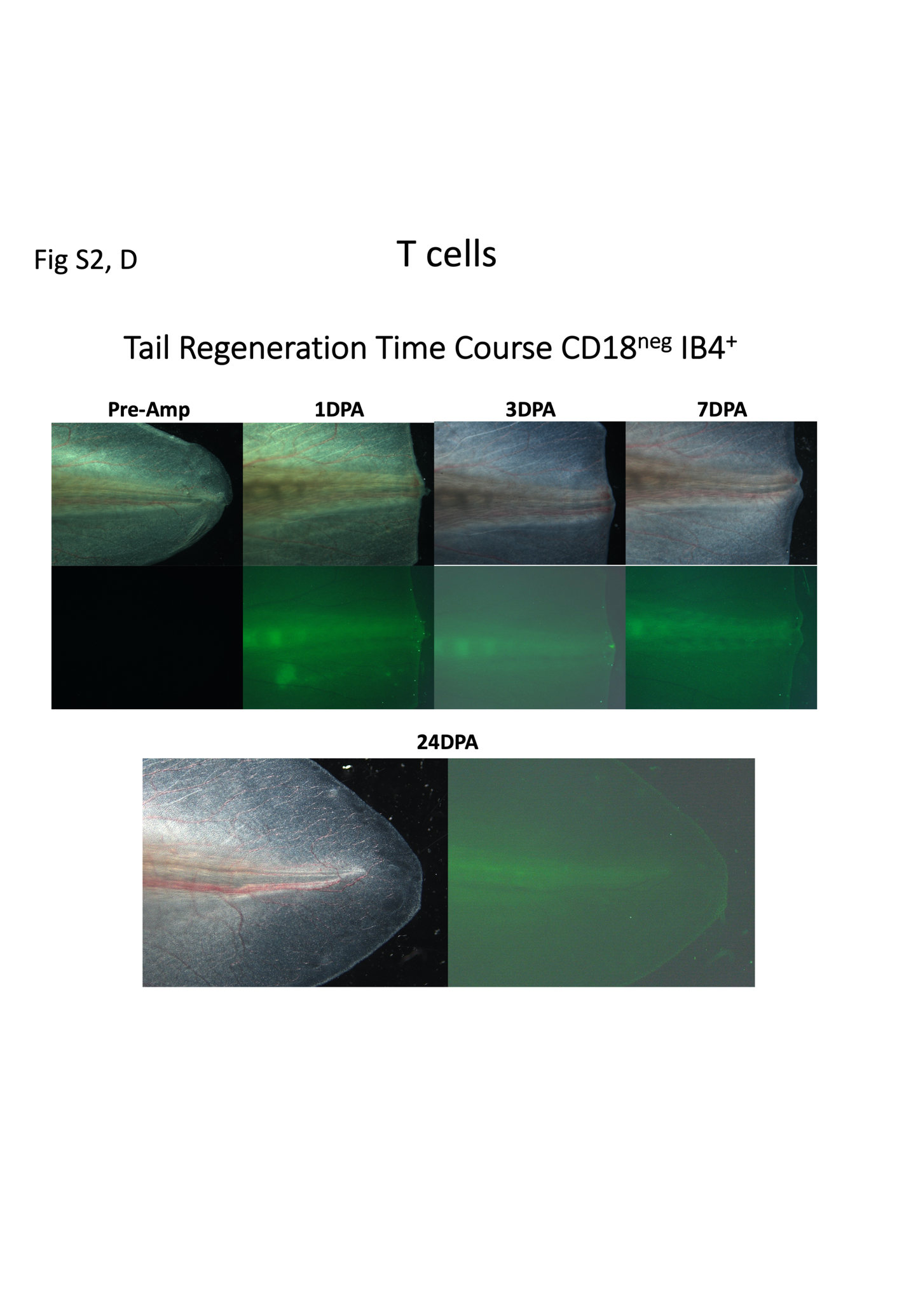
**

**Fig S2, (D)** Representative adoptive transfer experiments testing the potential for FACS sorted populations to traffic to amputated tails.Time course of T-cell (IB4^+^CD18^neg^) GFP^+^ donor cell recruitment at 1, 3 and 7 dpa days to the regenerating tail (N=5).

**
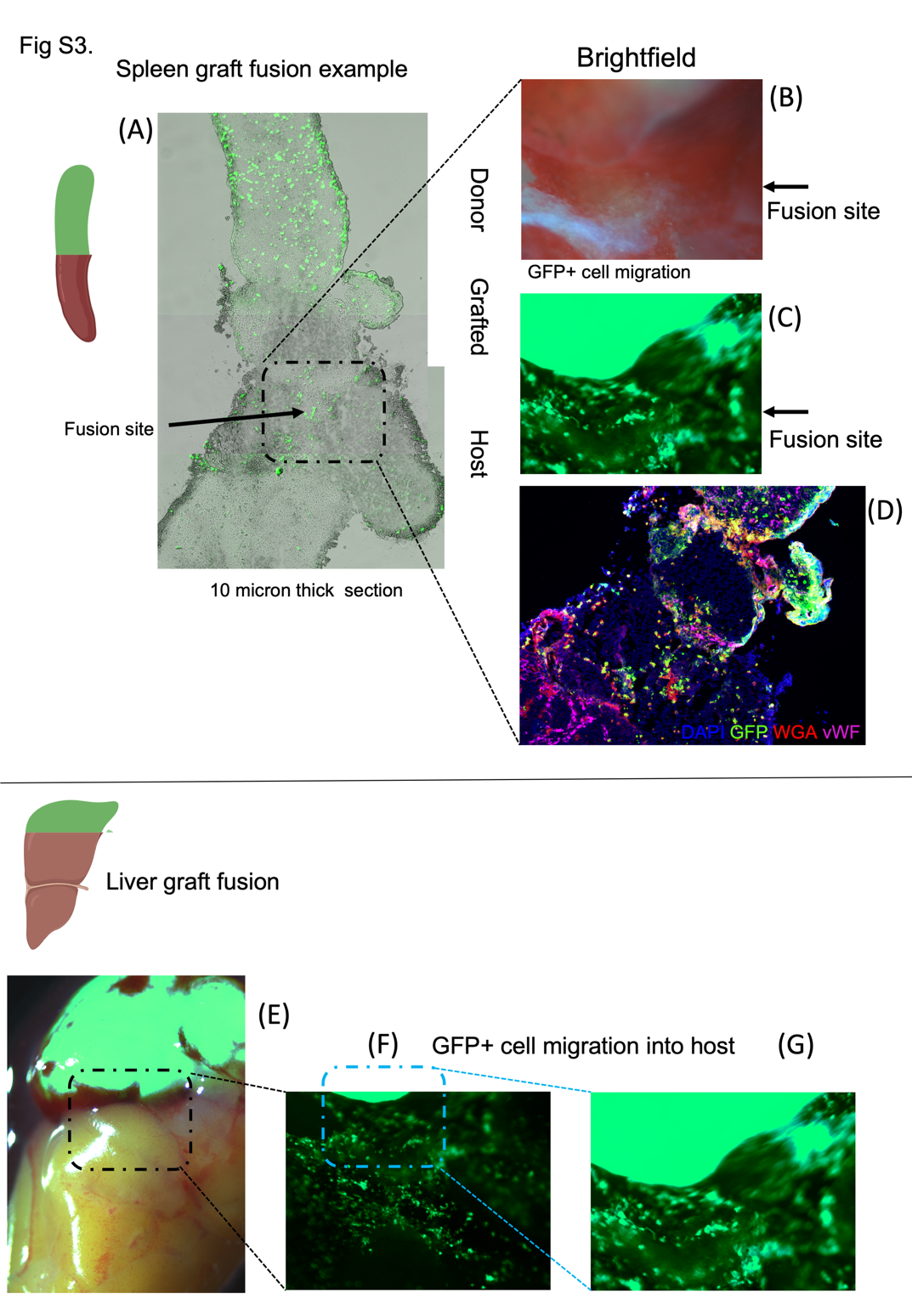
**

**Fig S3. GFP^+^ graft fusion of liver or spleens to d/d axolotl hosts.** **(A)** Tiled brightfield section showing an example of a join between host and donor liver. Note GFP ^+^cells invading the host non-GFP host. **(B)** Brightfield image on stereomicroscope showing tissue fusion. (**C)** Fluorescent imaging on stereomicroscope showing fusion region and migration of GFP+ cells into host tissue. **(D)** 10-micron thin section showing an example of GFP^+^ micration along von Willibrand (vWF) positive vasculature (magenta). Nuclei in Blue. Cell outlines stained in red with wheat germ agglutinin lectin (WGA).


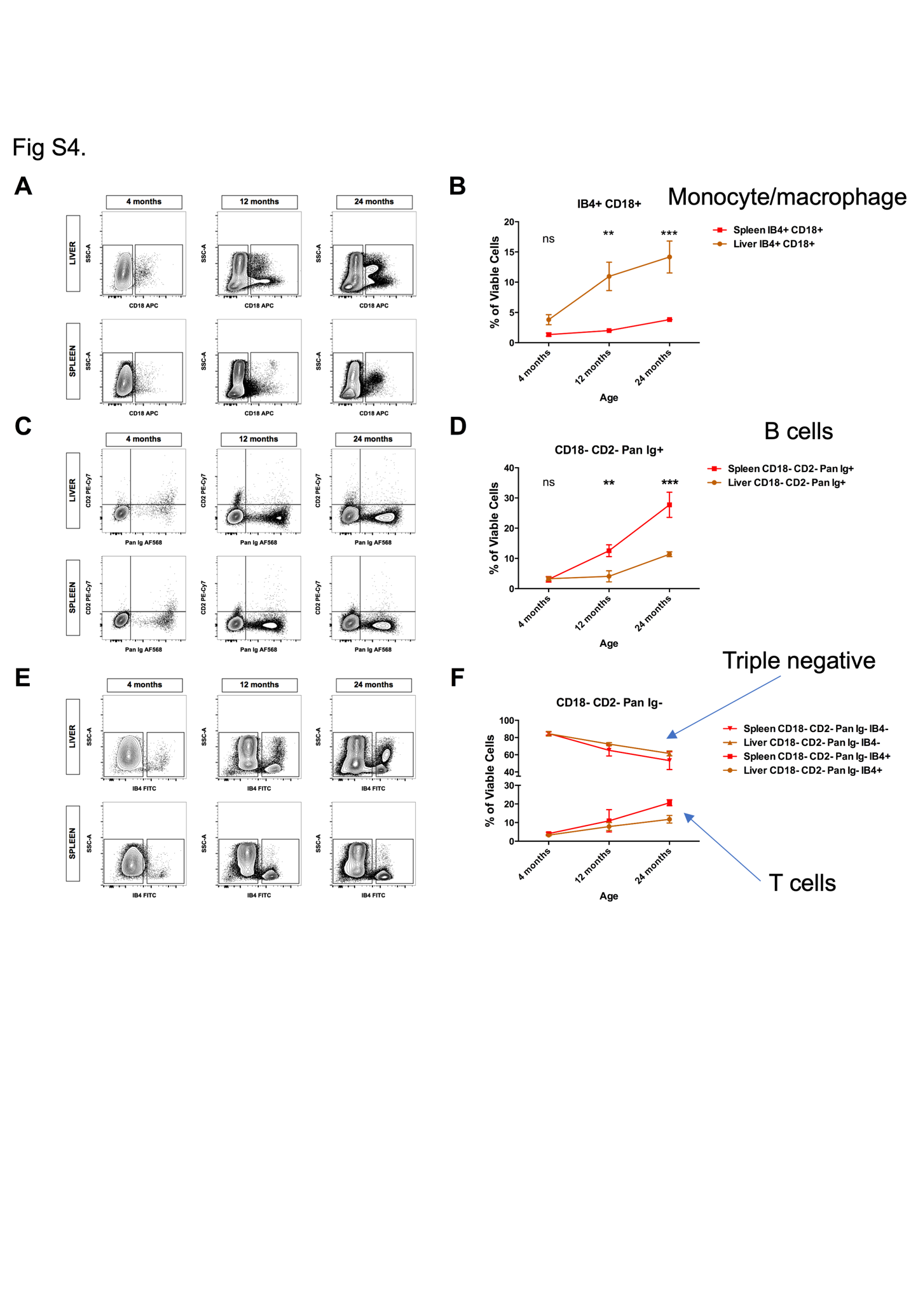


**Fig S4. Flow cytometry profiling of different leukocyte populations in liver and spleen at 4, 12 and 24-months old axolotls**. **(A, C, E)** Gating strategy for monocyte/macrophage (IB4^high^CD18^+^), B cells (PanIg^+^), Triple negative (IB4^neg^CD18^neg^PanIg^neg^) and T cell populations (IB4^+^CD18^neg^). **(B, D, F)** Comparative analysis of cell counts found in liver and spleen for each population. Error bars represent mean ± SEM of 3 animals per group at each time point. Adjusted p-values obtained via one-way ANOVA with multiple comparisons within each group. ** P ≤ 0.01, *** P ≤ 0.001, **** P ≤ 0.0001.


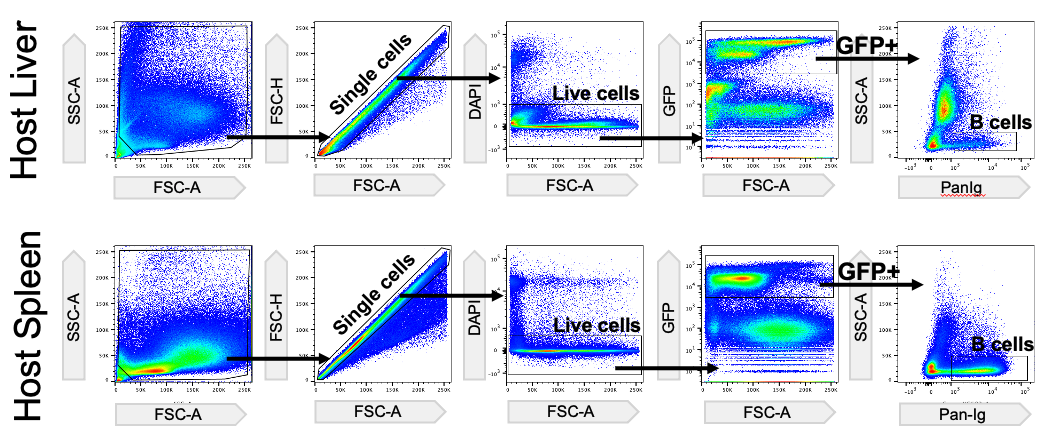


**Fig S5. Viable donor peripheral blood derived GFP+ B cells are found in host liver and spleens 15 days post adoptive cell transfer.** GFP+ donor blood cells were adoptively transferred into d/d white leucistic hosts. 15 days post transfer, spleen and liver tissues were harvested, dissociated, and stained with antibody panel. Flow cytometric staining of B cells using the axolotl specific PanIg antibody confirms the presence of live viable B cells for at least 15 days post adoptive transfer.

**Supplementary Table S1:**

| **Antibody/reagent** | **Clone** | **Application** | **Supplier (cat#)** |
| --- | --- | --- | --- |
| Isolectin B4-Biotin | N/A | FC (1 in 50) | Vector Labs (B-1205) |
| CD18 APC | TS1/18 | FC (1 in 100) | Biolegend (302114) |
| Pan Ig | N/A | FC (1 in 400) | Axolotl Monoclonal Antibody |
| CD20-AF700 | RM2-5 | FC (1 in 50) | Walter and Eliza Hall Institute |
| Streptavidin PE-Cy7 | N/A | FC (1 in 1000) | Biolegend (405206) |
| Goat anti-mouse IgG (H+L) AF568 | N/A | FC (1 in 1000) | Thermo Fisher Scientific (A11031) |
| CD2 Biotin | RM2-5 | FC (1 in 50) | Walter and Eliza Hall Institute |
| Pan Ig-PE | N/A | FC (1 in 400) | Axolotl Monoclonal Antibody |
| Isolectin B4-FITC | N/A | FC (1 in 50) | Vector Labs (FL-1201-.5) |
|  |  | FC: flow cytometry |  |

**Supplementary Table S2:** Primers for qPCR analysis

| **Primer** | **Sequence** |
| --- | --- |
| AxRBL27_F | CATCAGATCAAGCAAGCAGTA |
| AxRBL27_R | CCAATGCAGCAGTTTAGATG |
| AxBetaActin_F | TCCATGAAGGCTGCCCAACT |
| AxBetaActin_R | TGGCGCCACATCTGATTGAT |
| AxGAPDH_F | GACAAGGCATCTGCTCACCT |
| AxGAPDH_R | ATGTTCTGGTTGGCACCTCT |
| AxCSF1R_F | CTCCAGGATGGGACTGTCAT |
| AxCSF1R_R | CCGCTTGGAGGTAGAGTCTG |
| AxNeutrophil elastase | CGCCTCCCTACAGTTCAGAG |
| AxNeutrophil elastase | ATCGGACTCATTCCATCCAA |
| AxMPO_F | TCAACAGCTGGAGAATCGTG |
| AxMPO_R | ATGTTGATGGCGGCTAAATC |
| AxPRTN3_F | TGCAGCGGGCACCCAATGTG |
| AxPRTN3_R | GCCCGCCGGAATCCCAAAA |
| AxITGAM_F | GTGTCAACAAGGGTCGAGGT |
| AxITGAM_R | GCACTTCAGATGCAGGTTCA |
| AxPU.1_F | ATGCCTTTCCTGACAACCAC |
| AxPU.1_R | GCACATACGGGGCAAATAGT |
| AxCD3e_F | GGGGTCTCCATCTTGGTGTA |
| AxCD3e_R | GGCTCATAGTCTGGGTTTGG |
| AxTCRaC_F | GAAGGGAAAAGGGAAGCAAC |
| AxTCRaC_R | TGACTGCCATCACAGGACAT |
| AxPerorin_F | AGCTCATTGAGACCGCATTT |
| AxPerorin_R | CGCGGTGTGAAAGTTGTAGA |
| AxIGHM_F | CTGAACAGAGGGTGCTCTCC |
| AxIGHM_R | TTCTGCAGCTGCTGTGAGTT |
| AxIGLamConst_F | GGAGTTGAGACAGCCAAAGC |
| AxIGLamConst_R | TTGAGATCGGCTCACAGATG |
| AxRAG_F | AAGAATCCTGCGATGGAATG |
| AxRAG_R | AGCATGAGGCACAGAGGTTT |
